# Data set on work routine management among academic staff in selected Nigerian University: The eustress perspective

**DOI:** 10.1016/j.dib.2021.107621

**Published:** 2021-11-26

**Authors:** Adeshola Peter, Anthonia Adeniji, Kehinde Oladele, Fred Peter, Henry Inegbedion, Ademola Sajuyigbe

**Affiliations:** a Landmark University, Omu Aran, Nigeria; b Covenant University, Ota, Nigeria

**Keywords:** Academic, Cognitive appraisal, Demand management, Eustress, Performance, Nigeria, Strategy

## Abstract

Academics undergo stress, which negatively affects their performance and management does not seem to provide adequate organizational support for people suffering from occupational stress. This data captures eustress among university academic staff and coping strategies to improve performance. Previous studies on stress among academics focused on their experience of distress, with little attempt to explore eustress. particularly in the Nigerian context. A mixed method approach was adopted to elicit information from the sampled 444 respondents and twelve in-depth interviews were also conducted. Descriptive and inferential research methods were used for the analysis. The quantitative data were analyzed using Partial Least Square-Structural Equation Modelling (PLS-SEM), while the qualitative data were subjected to manual thematic analysis.

## Specifications Table

**Table:** 

Subject	Business, Management and Decision Sciences
Specific subject area	*Strategy and Management*
Type of data	Table Figure
How the data were acquired	Field Survey
Data format	Raw, analysed, Inferential statistical data
Parameters for data collection	*Different indicators of eustress were measured .in the study such as* Work routine management, Meeting research requirement Work routine management does not have significant impact on the community service engagement of academic staff. Cognitive appraisal strategy (positive mindset, locus of control, motivation, self-efficacy, experience, optimism, social support)
Description of data collection	Data was obtained from structured questionnaires administered to academic staff across six universities in Nigerian.
Data source location	• *Secondary data in supplementary Material* • *Institution*
Data accessibility	https://data.mendeley.com/datasets/gh9ynp4n49/1 V2, doi: https://doi.org/10.17632/gh9ynp4n49.2
Related research article	Iqbal, A., and Kokash, H. (2011), ‘Faculty perception of stress and coping strategies in a Saudi private university: An exploratory study,’ *International Education Studies, 4*(3), 137.

## Value of the Data

• These data explore the concept of eustress in enhancing the performance of academic staff. The data is valuable for configuring cognitive appraisal strategy that facilitates the development of a positive emotional state, enhances employee ability to make more novel connections and associations between ideas and creativity thereby improving productivity.
• This data shows the significance of work routine management which comprised skill discretion, meaningfulness, challenging task, management support and time management significantly and positively influence the performance of academic in the selected Nigerian universities.
• The data can be employed to enhance quality teaching research and community impact among academic staff thereby accelerating the attainment of world class status.
• The raw data is made publicly and allow policy makers and Federal Government Agencies such as Federal Ministry of Labor and Productivity, National Universities Commission (NUC) and Association of Vice Chancellors of Nigerian Universities (AVCNU), to formulate and implement policies, consistent with encouraging academic staff to maintain a positive emotion that will enable them experience eustress and guard against the negative consequences of stress.

## 1 Data Description

**Fig. 1:**
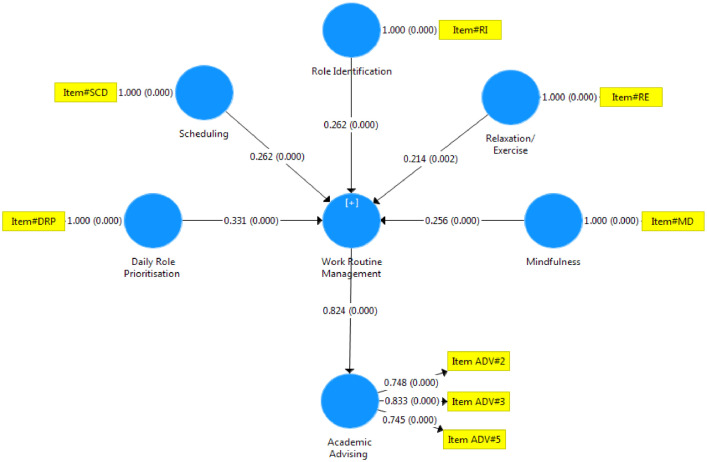
Predictive relevance (Path co-efficient) of Work routine management and Academic advising.

**Fig. 2:**
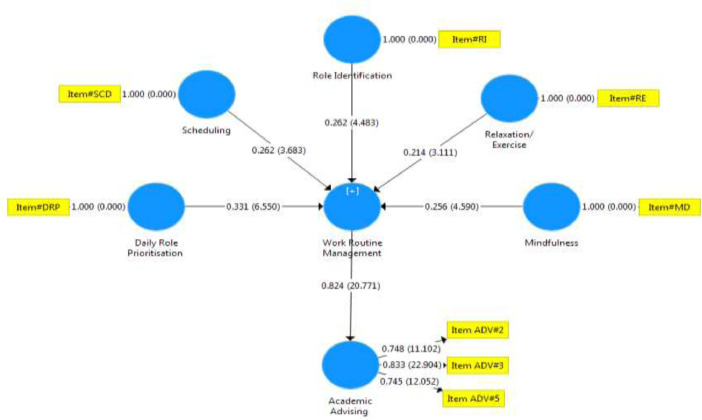
Path Co-efficient and T-values for Work routine management and Academic advising.

This article contains the Data compilation for the development of indicators of eustress and cognitive appraisal strategy that enhances academic staff performance. The data collected from the questionnaires administered to respondents were analysed and presented as follows. Table 1 the response rate from the questionnaire administered to the target respondents for the study, Table 2 indicates the breakdown of returned questionnaire. Table 3 depicted that all the constructs of the work routine management and Academic advising in the selected Nigerian universities have values higher than 0.80 and 0.70, which means that they have composite internal consistency and Cronbach Alpha reliability respectively, Figs. 1 and 2 respectively, depicted bootstrapping for work routine management and academic advising of universities was presented. Fig. 3 predicted that work routine management which comprised role identification, scheduling, documentation, prioritization, mindfulness and relaxation/exercise significantly and positively influence academic advising in the selected Nigerian universities. Table 4 indicated the path coefficient and bootstrapping of all constructs and significant relationships in the analysis at .05. Table 5. Shows the regression analysis establishing how an independent variable causes the dependent variable to change, and the results of the analysis are expected to change if independent and dependent variables are swapped. Table 6 shows that the RMSR of this model is 0.073, which is lesser than 0.08. This suggests that the RMSR for this model has a good fit

**Table 1:** Respondents’ response rate.

Distribution	Number	Percentage
Correctly filled and Returned	401	90%
Not Returned and not completely filled	43	10%
Total	444	100%

**Table 2:** Breakdown of retuned questionnaire.

S/N	University	University Code(s)	Copies Distributed	Copies Retrieved	% of Copies Retrieved
1	University of Lagos	Uni. A	80	74	92%
2	University of Ibadan	Uni. B	187	165	88%
3	Covenant University	Uni. C	32	31	97%
4	Babcock University	Uni. D	38	33	87%
5	Lagos State University	Uni. E	54	50	93%
6	Ladoke Akintola University of Technology	Uni. F	53	48	91%

**Total**			**444**	**401**	**90%**

**Table 3:** Factor loading for work routine management and academic advising.

	Factor Loading	Error Variance	Composite Reliability	AVE	Cronbach's Alpha	No. of Indicators
**Indicators**	**> 0.7**	**< 0.5**	**≥ 0.8**	**≥ 0.5**	**≥ 0.7**	

**Work Routine Management (WRM)**			0.843	0.554	0.820	5

WRM1	0.716	0.284				
WRM2	0.856	0.144				
WRM3	0.883	0.117				
WRM4	0.792	0.208				
WRM5	0.855	0.145				

**Academic Advising (AA)**			0.825	0.619	0.720	5

AA1	0.655	0.345				
AA2	0.748	0.252				
AA3	0.833	0.167				
AA4	0.619	0.381				
AA5	0.745	0.255

**Table 4:** Path coefficients for work routine management and academic advising.

Variables and Cross Loading	Path Co-efficient (O)	Indirect Effect (IE)	Std. Dev. (STDEV)	T-Statistics (O/STDEV		P Values
Daily role prioritization □ Work routine management	0.331		0.077	6.550		0.000
Daily role prioritization □ Academic advising		0.301	0.068	5.876		0.000
Scheduling □ Work routine management	0.262		0.061	3.683		0.000
Scheduling □ Academic advising		0.229	0.055	3.194		0.000
Role identification □ Work routine management	0.262		0.082	4.483		0.000
Role identification □ Academic advising		0.238	0.067	4.083		0.002
Relaxation/Exercise □ Work routine management	0.214		0.072	3.111		0.003
Relaxation/Exercise □ Academic advising		0.194	0.059	3.004		0.000
Mindfulness □ Work routine management	0.256		0.061	4.590		0.000
Mindfulness □ Academic advising		0.224	0.050	3.993		0.000
**Work routine management** □ **Academic advising**	0.824		0.073		20.771	0.000

	R Square (R^2^)		R Square (R^2^) Adjusted			
Work routine management □ Product Innovation	0.680		0.646

**Table 5:** Summary of regression for work routine management and academic advising.

Model Summary						
Model	R	R Square	Adjusted R Square	Predictive Value	t	Sig.
Work routine management	.824^a^	0.680	.646		20.771	0.000
Academic Advising _Uni. A	.466	0.217	0.204	0.259	4.284	0.000
Academic Advising _Uni. B	.489	0.239	0.216	0.272	4.722	0.000
Academic Advising _Uni. C	.564	0.318	0.307	0.313	5.474	0.000
Academic Advising _Uni. D	.522	0.272	0.266	0.290	5.153	0.000
Academic Advising _Uni. E	.517	0.267	0.256	0.287	5.026	0.000
Academic Advising _Uni. F	.405	0.164	0.151	0.225	4.027	0.000

**Table 6:** Model fit index for work routine management and academic advising.

Model Fit Index	Measures	Abbreviated	Accepted value	Model Value
Absolute Fit Index	The goodness of Fit Index	GFI	≥0.90	0.951
	Chi-square/Degree of Freedom	CMIN/DF	<3.0	2.527
	Root Mean Square Residual	RMSR	<0.08	0.073
Incremental Fit Index	Comparative Fit Index	CFI	≥0.90	0.939
	Normed Fit Index	NFI	≥0.90	0.920
Parsimony Fit Index	Parsimony Comparative Fit Index	PCFI	≥0.50	0.576

## 2 Experimental Design, Materials and Methods

Data was collected through the administration of structured copies of questionnaire to all academic staff in six universities across three states that are top ranking in Nigeria (2020 NUC Ranking) with teaching effectiveness, research and publication output, student enrolment, feasibility, student performance, quality graduate, technology, conducive learning environment, community service as indices for performance ranking amongst others (NUC, 2020). These are: University of Ibadan, Oyo State, University of Lagos, Lagos State, Covenant University, Ogun State, Babcock University, Ogun State, Lagos State University, Lagos State and Ladoke Akintola University of Technology, Oyo State. Emphasis was placed on six universities which are the first two top ranking public universities, the first two top ranking private universities and the first two top ranking state universities in terms of performance according to 2020 NUC Ranking. The emphasis on the academic staff of the organization was founded on the fact that academic staff of any university are the foremost crop of work force that drive performance hence, the concept of eustress may be more applicable to them. Regression analysis was employed to test the hypothesis stated. Eustress was measured using role demands management. Academic staff performance was assessed using a community service engagement. Responses ranged by 5-point Likert scaling from 1= “Strongly disagree” to 5= “Strongly agree. Data was also collected from secondary sources which involve relevant information based on published journals [1,2,[3], [4], [5], [6], [7]].

The response rate from the questionnaire given to the study's target respondents is shown in Table 1 The provided result was based on questionnaire responses that were correctly filled out and returned. The number of missing values on the research variables was minor and random, according to the missing data patterns. Further, imputation of missing values was not considered essential because the missing values were modest and randomly distributed, and missing values were removed pairwise in SPSS 26.0. This option only removes occurrences where one of the variables being linked or regressed has a missing value.

The data obtained from the selected academic staff (*faculty*) in South-west, Nigeria were presented using frequency distribution tables and inferential statistics. The selected Universities considered for this study are University of Lagos, University of Ibadan, Covenant University, Babcock University, Lagos State University and Ladoke Akintola University of Technology. A response rate of four hundred and one (401) copies of questionnaire was achieved, which accounted for 90% as depicted in Table 4.1.2. This response rate was achieved due to constant visitation to the selected universities and the inclusion of a well-trained research assistant. Efforts were also made to appeal to the selected academic staff to respond to the google form prepared and sent to ensure adequacy and representation. The result presented was based on the responses (via hard copies and google form) from the questionnaire that were correctly filled and returned. The probable reason for having 10% non-response rate could be due to COVID-19 pandemic lockdown.

Table 3 shows that all of the constructs of work routine management and Academic advising at the selected Nigerian universities have values greater than 0.80 and 0.70, respectively, indicating composite internal consistency and Cronbach Alpha reliability. The factor loadings for the construct specific measures ranged from 0.619 to 0.883. Because the primary condition for the degree of fitness was met satisfactorily, the instrument was deemed reliable and valid.

In the Partial Least Square, the standardized coefficient and path coefficients were calculated. The value was used to determine the significance of the hypothesis. The bigger the effect on the endogenous latent construct, the higher the value. However, in Figs. 2 and 3 respectively, bootstrapping for work routine management and academic advising of universities was presented.

This hypothesis predicted that work routine management which comprised role identification, scheduling, documentation, prioritization, mindfulness and relaxation/exercise significantly and positively influence academic advising in the selected Nigerian universities.

All of the constructs' path coefficients and bootstrapping indicate significant correlations in the study at .05. The model indicated statistically significant path co-efficient between daily role prioritization and academic advising (β=.301, T_val_ = 5.876, p=.000), the scheduling of the work routine management and academic advising (β=.229, _Tval_ = 3.194, p=.000); the role identification and academic advising (β=.238, _Tval_ = 4.083, p=.003); relaxation/exercise and academic advising (β=.194, _Tval_ = 3.004, p=.000); and mindfulness and academic advising (β=.224, _Tval_ = 3.993, p=.000). Hence, all the path coefficients were of practical importance since the significance level is below .05.

The result also suggested that daily role prioritization and role identification have the highest beta values among the constructs that best predict academic advising in the selected Nigerian universities; whereas, the relaxation/exercise had the least effect on academic advising in the selected Nigerian universities. Specifically, to determine and assess how work routine management effects academic advising in the selected Nigerian universities, path analysis and bootstrapping based on the institutional level were created. The structural models and path analysis for work routine management and academic advising based on universities demonstrated good predictive and explanatory ability.

The findings indicated a positive relationship between the work routine management and Academic advising in the selected Nigerian universities, as presented in Table 4.4.6. Regression analysis establishes how an independent variable causes the dependent variable to change, and the results of the analysis are expected to change if independent and dependent variables are swapped. Table 4.4.6 is a model fit that determines how the data fits with the model equation. The R^2^ was used to establish the study model variance of power.

All of the model fit indices were found to be within the acceptable range and above the specified cut-off level, according to the measurement model (Hancock and Mueller, 2018). The table shows that the RMSR of this model is 0.073, which is lesser than 0.08. This suggests that the RMSR for this model has a good fit. By implication, the null hypothesis one (H_01_) which indicates that the work routine management does not significantly have combined effects on academic advising in the selected Nigerian universities is hereby rejected. Above all, the results established that the work routine management is a significant predictor of academic advising in the selected Nigerian universities.

## Ethics Statements

As at the time this study was conducted ethics approval for the survey studies was not required. However, this study makes sure that the respondents had full disclosure of the nature of the study, the risks, benefits, and alternatives, with an extended opportunity to ask questions. Also, every respondent was presented with the opportunity to stay anonymous, and their responses were treated with the utmost confidentiality. All respondents were allowed to decline participation at will or respond to questions at any given time during the data collection process.

## CRediT authorship contribution statement

**Adeshola Peter:** Conceptualization, Methodology. **Anthonia Adeniji:** Supervision. **Kehinde Oladele:** Supervision. **Fred Peter:** Writing – original draft. **Henry Inegbedion:** Software. **Ademola Sajuyigbe:** Writing – review & editing.

## Declaration of Competing Interest

The authors declare that they have no known competing financial interests or personal relationships that could have appeared to influence the work reported in this paper.

## Appendix Supplementary materials

Image, application 1

Image, application 2
