# Supplementary material for: Data set on work routine management among academic staff in selected Nigerian University: The eustress perspective
Source: Data Brief. 2021 Nov 26;39:107621. doi: 10.1016/j.dib.2021.107621 (PMC8640227; doi:10.1016/j.dib.2021.107621)
Supplement: Supplementary file 1 [file mmc2.zip › mmc2/questionnaire on eustress.odt]

A picture containing text, clipart

Description automatically generated

              Department of Business Management  

  					              College of Management and Social Sciences 

 					              Covenant University, Ota, Nigeria 

              September 10, 2020 

Dear Respondent,

RESEARCH QUESTIONNAIRE 

I am a Ph.D student in the above named institution carrying out a research on “Organizational Eustress and Academic Staff Performance in Selected Nigerian Universities”. This study is being undertaken in partial fulfilment of the requirements for the award of Ph.D in Business Administration.

Please kindly fill in the correct information needed for the completion of this research. All information supplied will be used for the purpose of this study and will be treated with utmost confidentiality. 

Thank you. 

Peter, Adeshola O.

(Researcher)         

RESEARCH QUESTIONNAIRE 

DEPARTMENT OF BUSINESS MANAGEMENT, COVENANT UNIVERSITY, OTA, OGUN STATE

 

 Dear Respondent,

This questionnaire is based on a Ph.D dissertation exploring “Organizational Eustress and Academic Staff Performance in Selected Nigerian Universities”. I kindly ask for your voluntary co-operation in filling out this questionnaire. All information supplied will be used for the purpose of this study and will be treated with utmost confidentiality.  

 Thank you

SECTION A: GENERAL INFORMATION

INSTRUCTION: Kindly tick (✓) as appropriate and comment where necessary. 

- Name of University: (a) UNILAG [    ] (b) UI [   ] (c) CU [    ] (d) BABCOCK [    ] (e) LASU [    ] (f) LAUTECH [   ]

- Gender: (a) Male    [    ]	 (b) Female   [    ]

- Age: (a) 11 – 20 [    ] (b) 21 – 30 [    ] (c) 31 – 40 [    ] (d) 41 – 50 [    ] (e) 50 and above [    ]

- Highest Educational Qualification: (a) B.Sc. [  ] (b) M.Sc/MBA [  ] (c) M.Phil/Ph.D/Ph.D [   ] Others        (Please specify)_______________

- How long have you been lecturing at your university? (a) 1-5years [   ] (b) 6-10years [   ]               (c) 11-15years [    ] ( d) 16years and above [    ]

	

SECTION B: Kindly indicate by ticking (✓) as appropriate whether you “Strongly Agree (SA)”, “Agree (A)”, “Undecided (U)”, “Disagree (D)” or “Strongly Disagree (SD)”. 

| S/N | Questions | SA | A | U | D | SD |
| --- | --- | --- | --- | --- | --- | --- |
|  | Work Routine Management |  |  |  |  |  |
| 1 | My Job description is clear to me |  |  |  |  |  |
| 2 | I daily prioritise my responsibilities |  |  |  |  |  |
| 3 | My work schedule is well planned out |  |  |  |  |  |
| 4 | I keep an actionable to do list |  |  |  |  |  |
| 5 | I take time out to engage in relaxation and exercise |  |  |  |  |  |
|  | Academic Advising |  |  |  |  |  |
| 6 | I am able to create time to counsel students |  |  |  |  |  |
| 7 | Students benefit from my guidance |  |  |  |  |  |
| 8 | I help students to have clarity of academic goals |  |  |  |  |  |
| 9 | Students feel comfortable talking with me about concerns that pertain to academics |  |  |  |  |  |
| 10 | I motivate students to achieve their career goals |  |  |  |  |  |
|  | Meeting Research Requirement |  |  |  |  |  |
| 11 | I develop appropriate programme and time table for my student supervision |  |  |  |  |  |
| 12 | I create a scheduled plan for writing publication |  |  |  |  |  |
| 13 | My calendar is organised to accommodate conferences and workshops attendance |  |  |  |  |  |
| 14 | I am motivated to engage in partnership and collaboration with other faculty |  |  |  |  |  |
| 15 | I am able to meet the set publication criteria |  |  |  |  |  |
|  | Research Output |  |  |  |  |  |
| 16 | There is a high level of research productivity by academic staff |  |  |  |  |  |
| 17 | Academic staff are able to produce quality research |  |  |  |  |  |
| 18 | The research output of academic staff are of high impact |  |  |  |  |  |
| 19 | Large number of publications are turned out by academic staff |  |  |  |  |  |
| 20 | Universities have set up  research  department/ unit which  are  responsible for  coordinating  research  activities. Universities have set up  research  department/ unit which  are  responsible for  coordinating  research  activities. There is an effective research department in the university |  |  |  |  |  |
|  | Teaching Effectiveness |  |  |  |  |  |
| 21 | I am able to meet up with teaching obligation efficiently |  |  |  |  |  |
| 22 | Class sessions are attended to promptly |  |  |  |  |  |
| 23 | Teaching duties are discharged assiduously by academic staff |  |  |  |  |  |
| 24 | I diligently attend to lecture time |  |  |  |  |  |
| 25 | I have total control of my class |  |  |  |  |  |
|  | Role Demand  Management |  |  |  |  |  |
| 26 | My roles are performed skilfully |  |  |  |  |  |
| 27 | I positively perceive my role |  |  |  |  |  |
| 28 | My role is not demanding |  |  |  |  |  |
| 29 | There is provision for social support by management |  |  |  |  |  |
| 30 | I engage in time management to cope with the demand of my role |  |  |  |  |  |
|  | Community Service Engagement |  |  |  |  |  |
| 31 | Community service engagement is able to solve social problems in my community |  |  |  |  |  |
| 32 | The community service in my university is highly effective |  |  |  |  |  |
| 33 | Participation in developmental projects has impact in my community |  |  |  |  |  |
| 34 | The university immediate community benefit from community service |  |  |  |  |  |
| 35 | The university has an effective community service group/facilitators |  |  |  |  |  |
|  | Cognitive Appraisal |  |  |  |  |  |
| 36 | I am able to positively deal with the challenges of work routine |  |  |  |  |  |
| 37 | My years of experience help me to better deal with work stressors |  |  |  |  |  |
| 38 | I have control over how work stressors affect me |  |  |  |  |  |
| 39 | I anticipate positive event while coping with strain |  |  |  |  |  |
| 40 | I know what to do when I am under pressure |  |  |  |  |  |
|  | Eustress |  |  |  |  |  |
| 41 | I am able to effectively manage my work routine |  |  |  |  |  |
| 42 | I take time out to relax occasionally |  |  |  |  |  |
| 43 | The university’s set publication requirements are met by academic staff |  |  |  |  |  |
| 44 | I am able to cope with the demand of my roles |  |  |  |  |  |
| 45 | I can strategically cope with job stressors |  |  |  |  |  |
|  | Academic Staff Performance |  |  |  |  |  |
| 46 | There is provision of academic advising for students by academic staff |  |  |  |  |  |
| 47 | I impact my students through effective teaching |  |  |  |  |  |
| 48 | Quality and large number of research output is turned out by academic staff |  |  |  |  |  |
| 49 | I meet up with the timeline on my given task |  |  |  |  |  |
| 50 | I prepare lesson note regularly |  |  |  |  |  |

Universities have set up  research  department/ unit 

which  are  responsible for  coordinating  research  activities. 

Universities have set up  research  department/ unit 

which  are  responsible for  coordinating  research  activ
